# Colorectal cancer pre-diagnostic symptoms are associated with anatomic cancer site

**DOI:** 10.1186/s12876-024-03152-8

**Published:** 2024-02-06

**Authors:** Nicole L. Briggs, Mimi Ton, Rachel C. Malen, Adriana M. Reedy, Stacey A. Cohen, Amanda I. Phipps, Andrea N. Burnett-Hartman, Polly A. Newcomb

**Affiliations:** 1 Department of Epidemiology, University of Washington School of Public Health, UW Box #351619, 3980 15th Ave NE, Seattle, WA 98195 USA; 2 Public Health Sciences Division, Fred Hutchinson Cancer Center, 1100 Fairview Ave N, Seattle, WA 98109 USA; 3 Division of Oncology, University of Washington, 825 Eastlake Ave E, Seattle, WA 98109 USA; 4 Clinical Research Division, Fred Hutchinson Cancer Center, 1100 Fairview Ave N, Seattle, WA 98109 USA; 5 Institute for Health Research, Kaiser Permanente, 2550 S Parker Rd, Aurora, CO 80014 USA

**Keywords:** Prevention, Diagnosis, Red flag symptoms, Colorectal cancer, Age

## Abstract

**Background:**

Signs and red flag symptoms in colorectal cancer (CRC) patients who are below the recommended screening age are often overlooked, leading to delayed diagnosis and worse prognosis. This study investigates how patient pre-diagnostic symptoms are associated with anatomic site of their cancer and whether the association varies by age at CRC diagnosis.

**Methods:**

We ascertained CRC patients’ experienced symptoms and screening through medical abstractions from an ongoing population-based study of CRC patients identified through a SEER cancer registry (*N* = 626). We used logistic regression to estimate odds ratios and 95% confidence intervals for the association between symptoms and CRC anatomic site. Additional analyses were stratified by age at diagnosis. Early-onset was defined as less than 50 years of age at CRC diagnosis.

**Results:**

Participants who experienced blood in stool were more likely (odds ratio (95% confidence interval)) to have rectal (vs. colon) cancer (4.37 (3.02, 6.33)), as were patients who experienced changes to stool (1.78 (1.21, 2.60)). Patients diagnosed with colon cancer were more likely to present with abdominal pain (0.30 (0.19, 0.47)), anemia (0.40 (0.21, 0.75)), other symptoms (0.33 (0.19, 0.55)) and no symptoms (0.68 (0.44, 1.04)). When stratifying by age at diagnosis, we found that the association between blood in stool and rectal tumor location was particularly pronounced for patients with early-onset CRC (6.48 (2.73, 15.41)).

**Conclusions:**

Common pre-diagnostic red flag symptoms are associated with CRC anatomic site. These findings can inform best practices for gastroenterologist triage of care and early evaluation of CRC and are of key importance given the rise of early-onset (pre-screening age) CRC.

**Trial registration:**

Not applicable to this study and analysis.

**Supplementary Information:**

The online version contains supplementary material available at 10.1186/s12876-024-03152-8.

## Introduction

In the last few decades, incidence and mortality rates of colorectal cancer (CRC) have been decreasing in those over the age of 50, which can be attributed to increased screening rates [1]. There has been a rise of CRC cases in younger patients resulting in the recommended screening age being lowered to 45 years old in 2020 [2, 3]. However, national compliance for CRC screening is about 70%, which is below the national target of 80% [3, 4]. Even with screening, most CRC cases in the United States are diagnosed because of symptomatic presentation [5]. Recent evidence suggests that delays in referral and diagnosis for patients with symptomatic CRC is associated with a more advanced stage at diagnosis and a worse prognosis [6].

CRC patients younger than the recommended screening age without known risk factors are inherently diagnosed on diagnostic exams prompted by their symptoms rather than through routine, asymptomatic screening [7]. Often, signs and symptoms of CRC in these patients are underreported and overlooked despite their significant presence due to assumptions that these individuals are unlikely to have cancer [8]. Additionally, early-onset CRC patients tend to present with symptoms for a greater duration of time and/or intensity before diagnosis and may have more advanced stages of CRC at diagnosis compared to later-onset CRC patients [9]. Those with delays in cancer diagnosis have been shown to contribute to a poorer chance of survival [6].

Previous findings suggest that cancers in the proximal and distal sites of the colon may have different risk factors, but it is unclear if patients display pre-diagnostic symptoms that are specific to the anatomic site of their cancer and whether the association varies by age at CRC diagnosis. Red flag symptoms such as rectal bleeding, abdominal pain, change in bowel habits, unexplained weight loss, and anemia precede 70–95% of early-onset CRC cases [10]. A classic red flag symptom, rectal bleeding, is more likely to be associated with CRC in distal sections of the colon [11]. Thus, identification of red flag symptoms specific to CRC anatomic site could confirm appropriate triage for clinical care. This study aims to explore whether there is an association between CRC pre-diagnostic symptoms and anatomic site of the tumor, specifically between the colon and rectum. Identifying patterns of pre-diagnostic symptoms by anatomic site may provide future insight into best practices for early evaluation of CRC.

## Methods

### Study population

Participants were recruited through the Puget Sound Surveillance, Epidemiology and End Results (SEER) cancer registry. The Puget Sound SEER collects information on all cancers from residents across 13 counties in western Washington state. Participants in this study were diagnosed with CRC from April 1, 2016 to December 31, 2018 and were 20-74 years of age at the time of diagnosis [12]. Participants in the study were limited to those who were diagnosed with primary CRC.

We identified patients who had CRC during this period using International Classification of Diseases, Oncology, Version 3 (ICD-O-3) codes. There were 2,345 eligible CRC patients identified through SEER. Among those identified, 541 patients (23%) declined to enroll, 294 (13%) were lost to contact, and 56 (2%) were deceased. A total of 1,454 (62%) patients consented and were enrolled [12]. For this study, we excluded individuals for whom complete medical record abstraction was not available (*N* = 749). Individuals with hereditary CRC, diagnosed with inflammatory bowel disease, or who had a colectomy prior to their CRC diagnosis date were excluded (*N* = 69). Also excluded were those for whom no record data was available to determine whether symptoms vs. screening led to diagnosis (*N* = 10), leaving a final sample size of 626. A CONSORT diagram detailing the inclusion criteria appears in Additional File 1.

For this study, the Puget Sound SEER contacted eligible CRC patients via mail about 3 months post-diagnosis to inform them of their potential eligibility for research and to allow them to opt out of research contact. Patients who were alive at the start of their recruitment and who did not opt out of research were approached with an introductory study letter and a follow-up telephone call to assess study eligibility and address questions about their study participation and consent. Consenting participants completed a baseline questionnaire either by phone, on paper, or online. The questionnaire collected information on risk factors, general health, screening history, and demographics. The time from diagnosis to completing the questionnaire was on average 6.9 months (SD = 3.6). After questionnaire completion, participants could also provide written consent to release medical records to the study team for abstraction. All procedures were approved by the Institutional Review Board at the Fred Hutchinson Cancer Center [12].

### Assessment of screening or symptoms that led to diagnosis

For this analysis, we obtained information about screening and symptoms that led to diagnosis via medical record abstraction. Abstraction was performed by a trained abstractor. When the abstractor had questions regarding the interpretation of information, they met with the study team to reach a consensus. During data review, if data conflicted, a second abstractor performed quality control. The abstractor was asked to check all the following symptoms leading to the diagnosis that applied: blood in stool, changes to stool, abdominal pain or cramps, general weakness or constant fatigue, rectal fullness, gas, night sweats, unexplained weight loss, anemia, fever, vomiting, none, other, missing, and/or unknown. If no symptoms were mentioned as leading to diagnosis, there was a follow-up field where the abstractor entered the type of screening that led to diagnosis: fecal immunochemical test (FIT) or fecal occult blood test (FOBT), colonoscopy, sigmoidoscopy, rectal exam, barium enema, stool DNA test, other, missing, N/A, or unknown. For statistical analyses, guidance from a clinician determined how clinically similar symptoms were grouped. In particular, changes to stool, rectal fullness, and gas were grouped together under changes to stool. General weakness or constant fatigue, night sweats, weight loss, vomiting, and fever were grouped together under other symptoms.

### Colorectal cancer site

Tumor location was obtained for all participants from pathology reports through the Puget Sound SEER registry. Colon cancer was defined as ICD-O-3 codes C180 and C182–C189, and rectal cancer was defined as codes C199 and C209 [13].

### Covariates

Information regarding other health information and demographics was collected via the baseline questionnaire. We adjusted for the following variables known to be associated with the development and diagnosis of CRC: sex (male, female), age at diagnosis (years), race and ethnicity (people of color, white), diabetes (yes, no), and body mass index (BMI, kg/m^2^). We also adjusted for SEER cancer stage at diagnosis (localized, regional, distant), collected from the Puget Sound SEER to account for the fact that the stage of CRC may affect the presence of symptoms. In the absence of strong effect modification by cancer stage, this variable was included as a confounder.

### Statistical analysis

Logistic regression was used to calculate unadjusted and adjusted odds ratios (ORs) and 95% confidence intervals (CIs) to compare the odds of CRC site (rectal vs. colon) according to symptoms experienced. Symptoms were assessed as binary variables (yes, no). Logistic regression was performed on the following symptoms: blood in stool, changes to stool, abdominal pain or cramps, anemia, other symptoms, and asymptomatic.

An exploratory analysis was performed to analyze associations of CRC sites according to symptoms experienced stratified by age at diagnosis of CRC. Based on screening guidelines at the time of data collection, early-onset CRC was defined as those diagnosed with CRC before 50 years of age. Later-onset was defined as those who were diagnosed with CRC at age 50 years of age or older. In these exploratory analyses, we only adjusted for sex and BMI due to sample size constraints. In sensitivity analyses restricted to the later-onset group, there were no marked differences in results using this restricted adjustment model vs. the full adjustment model described above (see Additional File 2).

All statistical analyses were performed in SAS (Version 9.4, SAS Institute, Inc., Cary, NC, USA). All *p*-values were 2-sided and a *p*-value < 0.05 was considered statistically significant.

## Results

**Fig. 1:**
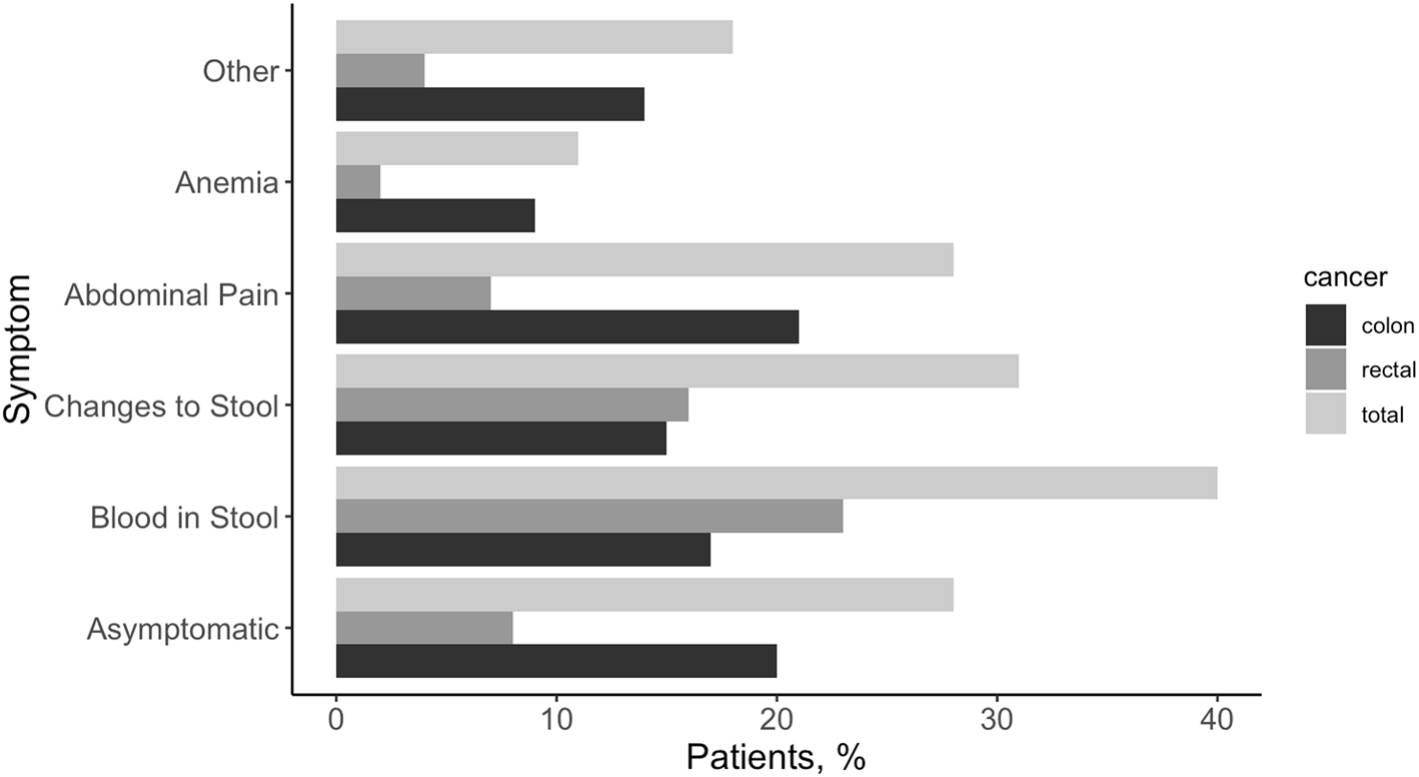
Percentage of patients who experienced symptoms by colon and rectal cancer (*N* = 626)

Among all CRC patients, 63% were diagnosed with colon cancer while 37% were diagnosed with rectal cancer. The majority of patients were white, had local or regional cancer stage at the time of diagnosis, and had an average age of CRC diagnosis of 58 years (Table 1). For the majority of patients (72%), cancer diagnosis was prompted by symptoms; those diagnosed based on symptoms were younger compared to those who were diagnosed through screening (average 57.0 vs. 61.8 years). The most prevalent symptoms were blood in stool (40%), changes to stool (31%), and abdominal pain (28%). Medical records also reported patients with anemia (11%) and other symptoms (18%) (Fig. 1). Asymptomatic patients who were diagnosed via screening received either a colonoscopy (83%) or FIT/FOBT (17%). While other screening tests were an option on the medical record abstraction form, none were noted.

**Table 1:** Characteristics of colorectal cancer patients (*N* = 626)

Characteristic	Total n(%)^b^	Diagnosed with Symptoms (*n* = 448) n(%)^b^	Diagnosed with Screening (*n* = 178) n(%)^b^
**Age at Diagnosis (years), mean (SD)**	58.4 ± 10.4	57.0 ± 10.9	61.8 ± 8.0
**BMI, mean (SD)**	27.8 ± 6.5	27.5 ± 6.2	28.7 ± 7.2
**Sex**			
Male	327 (52.2)	230 (51.3)	97 (54.5)
Female	299 (47.8)	218 (48.7)	81 (45.5)
**Race/Ethnicity**			
Caucasian/White	524 (83.7)	369 (82.4)	155 (87.1)
People of Color ^a^	84 (13.4)	64 (14.3)	20 (11.2)
**Cancer Stage**			
Localized	242 (38.7)	123 (27.5)	119 (66.9)
Regional	284 (45.4)	233 (52.0)	51 (28.7)
Distant	92 (14.7)	87 (19.4)	5 (2.8)
**Diabetes**			
Yes	93 (14.9)	61 (13.6)	32 (18.0)
No	531 (84.8)	387 (86.4)	146 (82.0)

^a^Includes African American/Black, Latino, Hispanic, or Spanish Origin, American Indian/Alaska Native, Asian, Native Hawaiian, or other Pacific Islander, Multiethnic

^b^Values may not add up to 100% due to missing data

The unadjusted and adjusted associations between CRC pre-diagnostic cancer symptoms and CRC anatomic site are reported and shown in Table 2 (reported as OR (95% CIs)). There were statistically significant associations between various symptoms and CRC anatomic site. CRC patients who experienced blood in stool (4.37 (3.02, 6.33)) and changes to stool (1.78 (1.21, 2.60)) were more likely to have rectal cancer than colon cancer. CRC patients with abdominal pain (0.30 (0.19, 0.47)), anemia (0.40 (0.21, 0.75)), or other symptoms (0.33 (0.19, 0.55)) were more likely to have colon cancer than rectal cancer. Asymptomatic patients (i.e. diagnosed via screening) were more likely to have colon cancer than rectal cancer (0.68 (0.44, 1.04)), although this result was not statistically significant.

**Table 2:** Adjusted odds ratios (OR) and 95% confidence intervals (CIs) for the association between each symptom and rectal anatomic site among patients diagnosed with colorectal cancer (*N* = 626)

Symptom	N (%)	Minimally Adjusted OR (95% CI)^ac^	Adjusted OR (95% CI)^bc^
Blood in Stool	250 (39.94)	4.08 (2.87, 5.80)^*^	4.37 (3.02, 6.33)^*^
Changes to Stool	193 (30.83)	1.91 (1.33, 2.73)^*^	1.78 (1.21, 2.60)^*^
Abdominal Pain	175 (27.96)	0.35 (0.23, 0.53)^*^	0.30 (0.19, 0.47)^*^
Anemia	70 (11.18)	0.44 (0.24, 0.81)^*^	0.40 (0.21, 0.75)^*^
Other Symptoms	114 (18.21)	0.41 (0.25, 0.66)^*^	0.33 (0.19, 0.55)^*^
Asymptomatic	178 (28.43)	0.68 (0.46, 1.00)	0.68 (0.44, 1.04)

^*^Statistically significant (*p*-value < 0.05)

^a^Adjusted for age at diagnosis (years)

^b^Adjusted for age at diagnosis (years), sex (male, female), diabetes (yes, no), BMI (kg/m^2^), cancer stage (localized, regional, distant), race/ethnicity (white, people of color)

^c^Most results remain statistically significant after a conservative Bonferroni correction for multiple comparisons

In exploratory analyses stratified by binary age at diagnosis, we observed that the associations between stool-based symptoms and rectal vs. colon cancer were stronger among those with early-onset CRC than among those with later-onset CRC (Table 3). Early-onset patients who experienced blood in stool were 6.48 (2.73, 15.41) times as likely to have rectal cancer while later-onset patients were 4.03 (2.71, 6.00) times as likely to have rectal cancer. Among early-onset patients, the OR for associations with rectal cancer site were 1.72 (0.80, 3.69) for changes to stool, 0.18 (0.08, 0.42) for abdominal pain, 0.19 (0.04, 0.94) for anemia, 0.18 (0.07, 0.48) for other symptoms, and 1.43 (0.22, 9.14) for no symptoms.

**Table 3:** Odds ratios (OR) and 95% confidence intervals (CIs) for the association between each symptom and rectal anatomic site among patients diagnosed with colorectal cancer, stratified by age at diagnosis (*N* = 626)

	**Early Onset (age < 50 years)** **(*n* = 116)**	**Later Onset (age ≥ 50 years)** **(*n* = 510)**
**Symptom:**	**Odds Ratio (95% CI)**^ab^	**Odds Ratio (95% CI)**^ab^
Blood in Stool	6.48 (2.73, 15.41)^*^	4.03 (2.71, 6.00)^*^
Changes to Stool	1.72 (0.80, 3.69)	1.87 (1.23, 2.85)^*^
Abdominal Pain	0.18 (0.08, 0.42)^*^	0.43 (0.26, 0.70)^*^
Anemia	0.19 (0.04, 0.94)^*^	0.48 (0.25, 0.94)^*^
Other Symptoms	0.18 (0.07, 0.48)^*^	0.46 (0.26, 0.83)^*^
Asymptomatic	1.43 (0.22, 9.14)	0.63 (0.42, 0.94)^*^

^*^Statistically significant (*p*-value < 0.05)

^a^All associations were adjusted for sex (male, female), and BMI (kg/m^2^)

^b^Most results remain statistically significant after a conservative Bonferroni correction for multiple comparisons

## Discussion

### Main findings

In this study, we observed statistically significant associations between CRC symptoms and CRC anatomic site. There was evidence that those who experienced blood in stool and changes to stool were more likely to be diagnosed with rectal cancer, while those who experienced abdominal pain, anemia, and other symptoms were more likely to be diagnosed with colon cancer. The observed positive association between the experience of blood in stool and rectal cancer site were particularly pronounced for early-onset CRC.

### Interpretation of findings

Rectal cancer patients diagnosed under the age of 50 (early-onset) are more likely to display symptoms for a length of time before they seek medical care perhaps due to a lack of risk awareness for their age or access to health services [10, 14]. Early onset rectal cancer patients are also more likely to present with a later disease stage [15]. Furthermore, there is an average 6-month time to diagnosis from symptom presentation in early-onset CRC patients [10]. Our study indicates that blood in stool is a more common pre-diagnostic symptom in patients who go on to a diagnosis of rectal vs. colon cancer. For early-onset CRC patients, the likelihood of being diagnosed with rectal cancer among those experiencing blood in stool increases. Because we know that patients with rectal cancer are more likely to display symptoms for a duration of time, this may differentially impact time to diagnosis in rectal cancer patients compared to colon cancer patients. The results also suggest that red flag symptoms may be predictive of early-onset rectal cancer. Specifically, blood in stool (a form of rectal bleeding) should be investigated further, as a common clinical scenario that is mistakenly attributed to hemorrhoids or other benign etiology and not generally evaluated further in a young patient.

### Implications for research and practice

The results of this study show that different symptoms portend a diagnosis of rectal vs. colon cancer. Despite increasing adherence to CRC screening and the revision of the recommended screening age to 45 years old in 2020, there are still frequent cases of CRC being diagnosed after the development of symptoms [2, 5]. Additionally, there has been an increase in CRC incidence rates in those less than the age of 50 who have symptomatic CRC [9].

Given that rectal cancer most often presents with red flag symptoms, blood in stool and changes to stool, clinical providers should pay acute attention to a patient's symptoms when ordering screening tests that detect CRC. Knowing what symptoms are more likely to be associated with rectal or colon cancer could result in more targeted diagnostic screening practices. Screening with a FIT/FOBT has been shown to be effective in reducing the mortality from rectal cancer but not in reducing the mortality from colon cancer [16]. FIT/FOBT is associated with early CRC diagnosis and may be an effective test for patients who are experiencing blood in stool or changes to stool [5].

### Strengths and limitations

There are a few limitations of this study that should be considered. Participants who did not have data for receiving a screening test or symptoms leading to diagnosis were excluded from the analysis due to missing exposure status, which limited our sample size. These participants were slightly older than the study population (60.5 years) and were mostly diagnosed with colon cancer. We also excluded participants with hereditary CRC, diagnosed with inflammatory bowel disease, or who had a colectomy prior to their CRC diagnosis date because they may have different screening recommendations or be at higher risk for CRC and should be analyzed separately from average-risk participants. The small sample size negatively impacted our CIs, potentially biasing results. We relied on medical record completeness and there may be some misclassification of symptoms and missed symptoms (due to incomplete patient recall or misunderstanding by the physician) which would attenuate our results. We also did not have information on levels of symptom severity or duration. Lastly, this study only included cases of CRC which prevents us from comparing the prevalence of symptoms among people who do not have cancer. To know if this association is significant in the general population, future research on this topic should consider including patients without cancer diagnoses and account for the time between symptom onset and diagnosis.

Some strengths of this study are that it is a well characterized population-based study. We used a broad and robust dataset that included medical record data and data from baseline questionnaires that allowed for adjustment of confounding variables, however residual confounding may still be present.

## Conclusions

Different pre-diagnostic symptoms are associated with rectal and colon cancer and some associations are stronger among early-onset CRC patients, specifically blood in stool. With the rise of early-onset CRC and with the recognition of suboptimal screening adherence in the general population, it is crucial for providers to consider CRC in the differential. This evaluation of red flag symptoms by anatomic site can be used to inform subsequent diagnostic evaluation, which may decrease the latency of CRC diagnosis in affected individuals and/or rule out cancer in unaffected individuals with similar symptoms.

### Supplementary Information

**Additional file 1.** Consort diagram detailing eligibility for the study, number of participants excluded, and final sample size.

**Additional file 2.** Comparing odds ratios (OR) and 95% confidence intervals (CIs) for the association between each symptom and rectal anatomic site to a sample restricted to those diagnosed with colorectal cancer ages 50 and older.

## Data Availability

Data, analytic methods, and study materials are publicly accessible to qualified investigators upon request to Amanda I. Phipps.

## Abbreviations

CRC: Colorectal Cancer
FIT: Fecal Immunochemical Test
FOBT: Fecal Occult Blood Test
SEER: Surveillance, Epidemiology and End Results
ICD-O-3: Classification of Diseases, Oncology, Version 3
