# Supplementary figures and images for: Colorectal cancer pre-diagnostic symptoms are associated with anatomic cancer site
Source: BMC Gastroenterol. 2024 Feb 6;24:65. doi: 10.1186/s12876-024-03152-8 (PMC10845784; doi:10.1186/s12876-024-03152-8)

Additional file 1

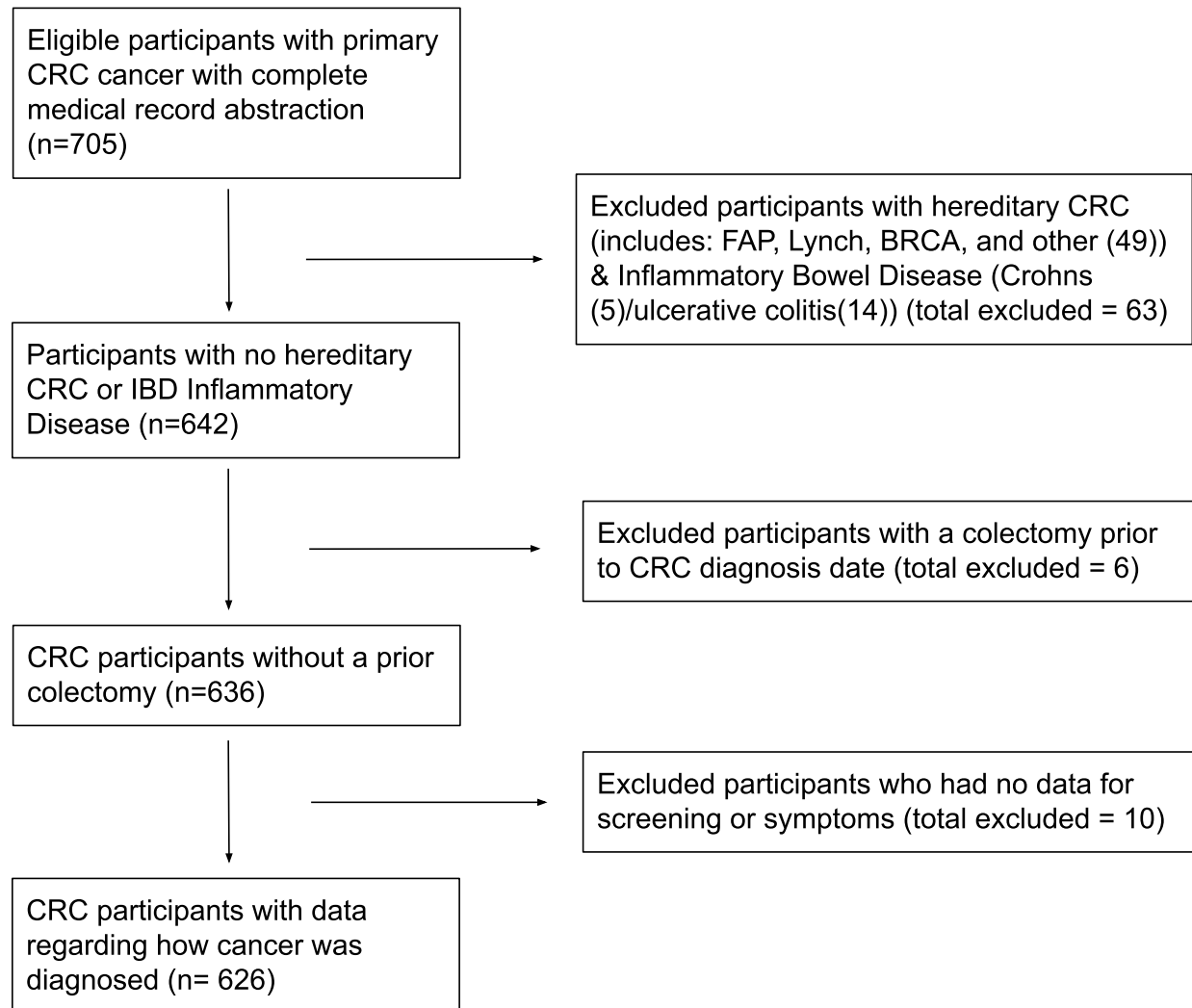

Supplement: Supplementary file 1 — Additional file 1. Consort diagram detailing eligibility for the study, number of participants excluded, and final sample size. [file 12876_2024_3152_MOESM1_ESM.pdf]
