# Supplementary material for: Colorectal cancer pre-diagnostic symptoms are associated with anatomic cancer site
Source: BMC Gastroenterol. 2024 Feb 6;24:65. doi: 10.1186/s12876-024-03152-8 (PMC10845784; doi:10.1186/s12876-024-03152-8)
Supplement: Supplementary file 2 — Additional file 2. Comparing odds ratios (OR) and 95% confidence intervals (CIs) for the association between each symptom and rectal anatomic site to a sample restricted to those diagnosed with colorectal cancer ages 50 and older. [file 12876_2024_3152_MOESM2_ESM.pdf]

Additional file 2

	All ages (N = 626)	Age ≥ 50years (N = 510)
**Symptom**	**Adjusted OR (95% CI)[a]**	**Adjusted OR (95% CI)[a]**
Blood in Stool	4.37 (3.02, 6.33)*	3.86 (2.55, 5.84)*
Changes to Stool	1.78 (1.21, 2.60)*	1.81 (1.15, 2.83)*
Abdominal Pain	0.30 (0.19, 0.47)*	0.38 (0.22, 0.63)*
Anemia	0.40 (0.21, 0.75)*	0.49 (0.24, 0.98)*
Other Symptoms	0.33 (0.19, 0.55)*	0.45 (0.24, 0.83)*
Asymptomatic	0.68 (0.44, 1.04)	0.62 (0.39, 0.97)*

[a]Adjusted for age at diagnosis (years), sex (male, female), diabetes (yes, no), BMI (kg/m^2), cancer stage (localized, regional, distant), race/ethnicity (white, people of color)
*Statistically significant (p-value < 0.05)
